# From Devo to Evo: patterning, fusion and evolution of the zebrafish terminal vertebra

**DOI:** 10.1186/s12983-020-00364-y

**Published:** 2020-06-01

**Authors:** Nicolás Cumplido, Miguel L. Allende, Gloria Arratia

**Affiliations:** 1 FONDAP Center for Genome Regulation, Facultad de Ciencias, Universidad de Chile, Santiago, Chile; 2 University of Kansas, Department of Ecology and Evolutionary Biology, Biodiversity Institute, Lawrence, KS USA

**Keywords:** Evo-Devo, Axial skeleton, Ural region, Pleurostyle, Compound centrum, Autocentrum, Chordacentrum, *Danio rerio*

## Abstract

**Background:**

With more than 30,000 species, teleosts comprise about half of today’s living vertebrates, enriched with a wide set of adaptations to all aquatic systems. Their evolution was marked by modifications of their tail, that involved major rearrangements of the metameric organization of the axial skeleton. The most posterior or ural caudal skeleton, primitively included more than 10 vertebrae and, through a series of fusions and losses, became reduced to a single vertebra in modern ostariophysans, one of the largest clades of teleosts. The ontogeny of the ostariophysan *Danio rerio* recapitulates this process by forming two or three separate vertebrae that become a single vertebra in adults. We characterize the developmental sequence of this change by describing the processes of patterning, fusion and differential growth on each of the constitutive elements that sculpt the adult terminal vertebra.

**Results:**

The ontogenetic changes of the terminal vertebra were characterized, highlighting their shared and derived characters in comparison with other teleosts. In zebrafish, there is: i) a loss of the preural centrum 1, ii) the formation of an hourglass-shaped autocentrum only in the anterior but not the posterior border of the compound centrum, iii) the formation of a vestigial posterior centrum that does not form an autocentrum and becomes incorporated beneath the compound centrum during development, and iv) the elongated dorso-posterior process of the compound centrum or pleurostyle appears as an independent element posterior to the compound centrum, before fusing to the ural neural arches and the anterior portion of the compound centrum.

**Conclusions:**

The unique features of the formation of the terminal vertebra in *Danio rerio* reflect the remarkable changes that occurred during the evolution of teleosts, with potential shared derived characteristics for some of the major lineages of modern teleosts. A new ontogenetic model is proposed to illustrate the development of the terminal vertebra, and the phylogenetic implications for the evolution of caudal skeleton consolidation in ostariophysans are discussed.

## Background

**Fig. 1:**
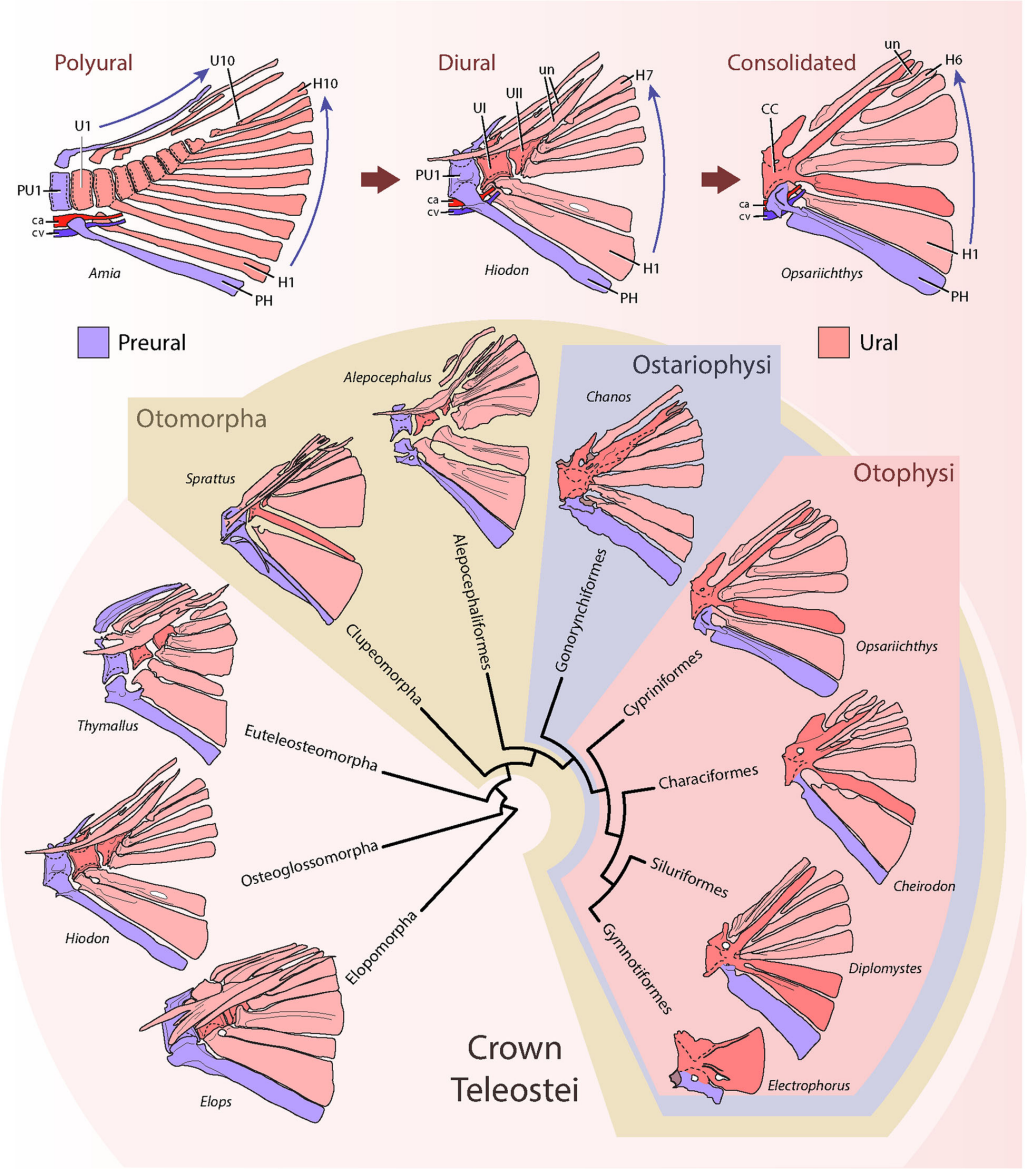
Evolution of the terminal vertebrae among teleosts. The phylogeny shows the main clades of basal teleosts focusing in ostariophysans, with a representative basal member of each group showing the posterior vertebra(e) that bears the hypural series (in red) and the parhypural (in purple). The phylogeny was adapted from Arratia (1999) and Betancour et al. (2017). Terminology: ca, caudal artery; CC, compound centrum; cv, caudal vein; H1–10, hypural series; haPU1, haemal arch of the preural centrum 1; PH, parhypural; PU1, preural centrum 1; U1–10, ural centra, Un; uroneurals

The teleosts, with more than 30,000 species, comprise the largest and most diverse clade of ray-finned fishes or Actinopterygii, dominating worldwide marine and freshwater systems [1]. Their evolutionary history was marked by several novelties [2], including significant changes on the postcranial skeleton [3–7], particularly, the evolution of a homocercal tail [4, 5, 8–13]. These changes involved a rearrangement, reduction and loss of several bones, including a reduction in the number of vertebrae articulating with the caudal fin rays, as well as the modification of the terminal neural arches into a series of longitudinally elongated bones known as uroneurals [8, 9, 11, 14]. This trend towards a more consolidated caudal skeleton generated a wide set of characters in each of the major branches of extant teleosts (Fig. 1), which captivated the attention of early morphologists due to its possible systematic value for the elucidation of the relationships of modern and fossil teleosts (e.g. [9, 14–24]).

A breakthrough in the comparative analysis of the teleostean caudal endoskeleton (which includes the last vertebral centra plus their modified elements) was the adoption of Nybelin’s convention [20], that allowed a common reference system for the analysis of the caudal fin vertebrae among multiple taxa, by discriminating between two distinct anatomical regions on the posterior axial skeleton, an anterior *preural* and a posterior *ural*, separated by the exit point of the main caudal artery and main caudal vein from the last haemal arch of the axial skeleton (ca & cv, Fig. 1). The ural region is a highly modified region, where, generally, the notochord flexes dorsally during development to form the homocercal tail, also characterized by their progressively smaller vertebral centra numbered from anterior to posterior (U1, U2, U3 …) and an absence of haemal arches. Ventrally it supports a series of modified unpaired haemal spines or hypurals, generally distally expanded, while dorsally it may support the uroneurals, modified ural neural arches extending longitudinally over the last vertebral bodies, which may increase the overall stiffness of the caudal skeleton. The preural region involves a series of less modified vertebrae than the urals, numbered from posterior to anterior (… PU3, PU2, PU1), generally having neural and haemal arches that, through their dorsal and ventral spines, support the procurrent rays of the caudal fin and/or the last principal rays The preural region shows transitional features between the more anterior caudal vertebrae and the ural region, particularly evident in its most posterior element, the preural centrum 1 (PU1), that bears a distally expanded haemal spine resembling a hypural and referred later as the parhypural (PH) [21]. In the polyural caudal skeleton of *Hiodon*, *Elops* and studied salmonids (e.g. [8, 10]), the last ural vertebrae have a reduced contribution of intramembranous ossification of the centrum compared to more anterior vertebrae. Therefore, the centra are progressively smaller caudally, but do not retain the exact width of the notochord, since their perichordal ossification still produce a thickening, particularly on the ventral portion of the centra [8, 10].

By comparing the caudal skeleton of actinopterygian fishes through this convention, two common patterns were characterized: a *polyural* and a *diural* pattern [25] (Fig. 1). In the *polyural* pattern, considered the primitive condition for teleosts and present in extinct basal groups such as †Pholidophoriformes, each ural vertebral body or centrum bears a single hypural and a single neural arch in a 1-to-1 relationship or metameric pattern [9, 14, 25, 26]. On the other hand, in the derived *diural* pattern, present in extant teleosts, these 1-to-1 relationships were lost, and the adult caudal skeleton generally comprises two ural centra (U1 and U2^D^), the first of which supports the lower hypurals (H1 and H2), and the second supports the upper hypurals (see e.g.: *Elops*, *Hiodon*, *Thymallus, Sprattus, Alepocephalus*; Fig. 1) [8–11, 27]. This transition from *polyural* to *diural* is also observed during the ontogeny of modern teleosts, for example in *Hiodon*, *Elops* and salmonids [8, 10], where distinct and separate vertebral centra may form at the base of the hypurals in a sequential and metameric pattern.

A second major modification of the caudal skeleton occurred in more advanced teleosts, where all of the hypurals plus the parhypural articulate into a single terminal or compound centrum (CC). This kind of centrum was convergently acquired among advanced forms of extant groups of teleosts, including derived clupeomorphs, several groups of euteleosts, and all living ostariophysans [11]. This latter group is of special attention due their rich biodiversity and the variation in the patterns of fusion between the compound centrum and the hypurals in each of its major lineages (Fig. 1) [9, 15, 16, 22, 28–30]. Among them, the cypriniform *Danio rerio* shares a remarkably similar caudal skeleton to the hypothesized primitive condition of otophysans, the major clade of Ostariophysi (represented by the cypriniform *Opsarichthys*: see Fig. 1) [15, 29]. Therefore, the study of its skeleton will allow us to understand the evolution of the compound terminal vertebrae. In this context, the zebrafish caudal skeleton includes the presence of the following combination of characters: a compound terminal centrum fused ventrally with the hypural 2 (H2), ventrally articulating but not fusing to the hypural 1 (H1) plus the parhypural (PH), while dorsally bearing a single neural arch plus the pleurostyle, with a single autogenous uroneural lying on its posterior portion [11, 13, 15, 28–31].

Several questions remain about the developmental and evolutionary processes leading to the formation of the single compound terminal centrum in ostariophysans, including its fusion to the hypurals and the pleurostyle [11, 27]. Among them, there is the ontogenetic pathway leading towards the formation of the vertebral centrum that involves the association throughout development between an *inner* mineralization of the middle layer of the notochordal sheath to form a ring-like chordacentrum [6, 32–34] and an *outer* ossification outside of the external notochordal sheath to form an hourglass-shaped autocentrum [6, 11, 33, 35–37]. Also, there is the question on the homology of the elements that form the terminal vertebra, which is key to allow a proper comparison between the caudal skeleton of different teleosts [9, 11, 13]. To date, the field has suffered from a disparate nomenclature that affects the interpretation of their evolution [11], particularly evident in recent analyses of zebrafish [11, 13, 29–31]. These interpretations involve assumptions taken during the identification of the elements that appear during ontogeny. For example, for zebrafish, the *diural* interpretation assumes that the two or three centra that appear during development correspond to the condition observed in *adults* of basal teleosts, which have a preural centrum 1 (PU1), a first ural centra (U1^D^) and a second ural centrum (U2^D^) [31]. On the other hand, the *polyural* interpretation compares these centra with the condition observed in *embryos* of basal teleosts, in which their development can be traced back to a particular segment on the caudal skeleton [11, 13].

Thus, two main goals were sought in this work. The first was to provide new and detailed morphological descriptions on the ontogeny of the compound terminal centrum, in order to analyze and contrast previous descriptions of its metameric or segmental organization. Therefore, the ural centra will be analyzed using a polyural nomenclature, referring to them with the superscript “P”, in contrast to the diural nomenclature of previous works [30, 31], referred to by the superscript “D”. The second goal was to evaluate the fusion events that occur during the assembly of the centrum, which could help to explain the diversity of fusion patterns between zebrafish and other ostariophysans. A novel ontogenetic model was generated to clarify the results and provide a clear basis for a detailed phylogenetic comparison.

## Materials and methods

### Zebrafish husbandry and experimental conditions

Zebrafish embryos were collected by natural spawning in our facility and raised at 28 °C in E3 medium (5 mM NaCl, 0.17 mM KCL, 0.33 mM CaCl2, 0.3 mM MgSO4, and 0.1% methylene blue, buffered at pH 7.0) in Petri dishes, according to standard procedures [38]. Feeding larvae and juveniles were raised up to one-or-two-months post-fertilization or until adulthood, under 14:10 light-dark cycle conditions, at a density of 20 fish per liter, and fed twice or three times a day with dry particularized food (Gemma, Skretting, Norway). Water conditions were kept constant at 28 °C, pH 7–7.3 and 600–800 μS. Adult fish were maintained at a density of 4–8 fish per litre and fed twice a day. Embryonic, larval and juvenile stages are expressed in hours post-fertilization (hpf), days post-fertilization (dpf), or by standard length (SL). All fish manipulations were performed under anesthesia with MS-222 (Tricaine, A5040, Sigma-Aldrich, MO, USA). Animal procedures and protocols complied with guidelines and had the approval of the Animal Ethics Committee of the University of Chile.

### Staging and measurements of zebrafish juveniles and adults

To search for the time points when the zebrafish terminal centrum develops, we examined the external anatomy and length based on standard ontogenetic tables [39], and the published sequence of development and ossification of the axial skeleton [31]. Measurements were taken as notochord length (NL) for pre-flexion larvae, or standard length (SL) for post-flexion larvae, juveniles and adults considering the length from the tip of the snout until the posterior end of the notochord (NL) or the posterior margin of dorsal hypurals (SL).

### Clearing and staining

More than 200 specimens, including a developmental series ranging from 4 to 32 mm SL were cleared and stained with alizarin red and alcian blue. The method used was slightly modified from that previously described [10, 40–42], including: 1) zebrafish larvae and adults were first euthanized and fixed in 70% ethanol; adults were then degreased in 95% isopropanol for several days, and transferred back to 70% ethanol before staining. 2) Alcian blue solution was prepared at 0.1% and dissolved in 70 to 30% ethanol / glacial acetic acid and stored for at least 4 to 5 months before using, which probably increased its pH, although it was not measured. 3) Larvae and adults were cleared for several days in saturated borate solution after alcian blue staining (without an incubation in trypsin), and then post-fixed in 4% buffered formaldehyde solution for 20 min to 1 h, depending on fish size. 4) Specimens were bleached slowly by adding a few drops of 3% H_2_O_2_ per 20 mL of 1% KOH. 5) Alizarin red staining and glycerol clearing was performed as previously described [10]. This material is currently stored at the Developmental Biology laboratory at the Faculty of Sciences of the University of Chile (uncatalogued).

During the analysis of zebrafish morphology, this work also considered specimens originated from other laboratories and previously prepared through other variants of the clearing and staining procedure [11, 13], currently stored at the Division of Fishes of the Museum of Natural History of the University of Kansas (KU:KUI), catalogued under the numbers KU:KUI 29144, 3 specimens of about 25.4 and 30.6 mm SL; KU:KUI 40245, for a day-to-day ontogenetic series of about 100 specimens between 6 to 27.9 mm SL; and KU:KUI 41369 to 41,370, 12 specimens of 5.3 to 6.8 mm SL.

### Generation of stable transgenic lines

To study the pattern of osteoblast localization during development, a stable transgenic line was generated by injecting the transgenic construct *osterix*:nuGFP using the Tol2 transposon system into one-cell stage zebrafish embryos [43] and screened for at least two generations. This construct was kindly provided by Prof. Stefan Schulte-Merker (Münster University) and contains the upstream regulatory region of the medaka *osterix*/*sp7* gene [44].

### In vivo skeletal staining

The pattern of bone deposition was assessed through the Alizarin Red S vital or live staining technique, as previously described [45]. Briefly, during every round of staining, 30 to 40 larvae or juveniles were transferred into petri dishes and immersed into a 0.01% ARS solution (Alizarin Red S, A5533, Sigma-Aldrich, MO, USA) diluted in system water and buffered at pH 7.5. Incubation times varied between 10 to 20 min depending on fish size. Afterwards, fish were rinsed three to six times in system water, anesthetized and visualized under a fluorescence stereomicroscope. Four or five specimens from each batch were selected and mounted in 0.7% agarose dissolved in E3 solution, before visualizing under the confocal microscope.

### Image acquisition and processing

Confocal images were acquired using a Zeiss LSM 510 and 710 and ZEN software. Images were processed in Fiji. Figures were assembled using Adobe Photoshop and Adobe Illustrator. Confocal images were post-processed to generate 3D rendering models by the VGStudio MAX software.

## Results

### Adult morphology of the zebrafish caudal fin skeleton

**Fig. 2:**
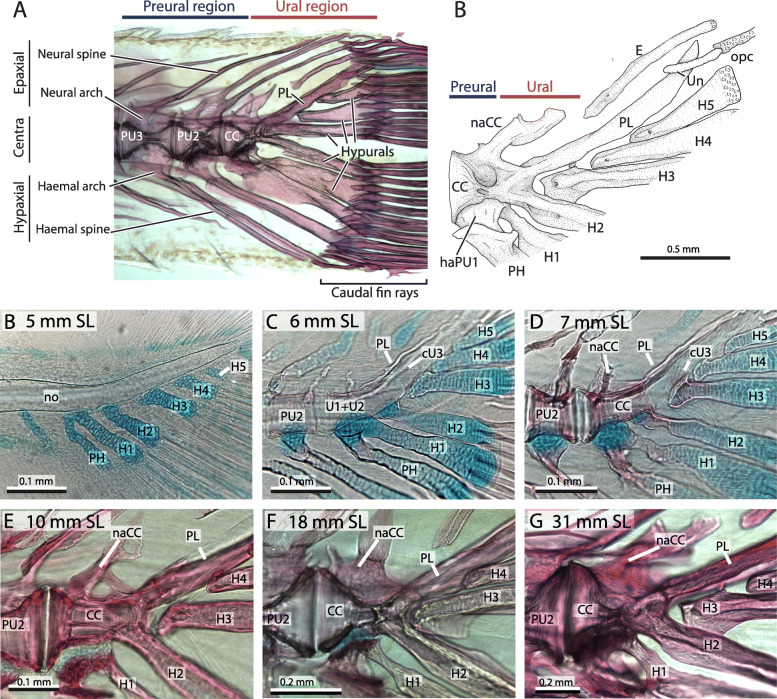
Adult morphology of the zebrafish caudal fin skeleton**. A**. A 22 mm SL zebrafish, showing the differences between the preural and ural region. **B**. Diagram of the terminal compound centrum of a 31.92 mm SL zebrafish illustrating its preural-ural boundary. **C-H**. series of cleared and stained zebrafish showing the main changes of the compound terminal vertebra. Terminology: CC, compound centrum; cU3–4, ural chordacentra 3 and 4; E, epural; H1–5, hypurals 1 to 5; haPU1, haemal arch of the preural centrum 1; hy, hypurapophysis; naCC, neural arch of the compound centrum; PH, parhypural; PL, pleurostyle; PU2, preural centrum 2; Un, uroneural

The adult morphology of the zebrafish caudal fin skeleton has been described previously [29, 31]. It consists of three vertebrae, two preural (PU3 and PU2) plus the terminal compound centrum (CC). The preural centra 3 and 2 have an amphicoelous or hourglass shape, fused dorsally with the neural arches and ventrally with the haemal arches, that extend distally through unpaired spines, articulating with the outermost procurrent caudal fin rays. The terminal compound centrum has a half hourglass shape, with its broad portion adjacent to the PU2 (Fig. 2b). This portion articulates ventrally with the last preural element, the haemal arch of the parhypural (PH), which is fused with the base of the hypural 1 (H1). Dorsally lies a single and short neural arch (naCC), highly variable in shape and size among all specimens analyzed. On its posterior margin, the compound terminal centrum is fused dorsally to the pleurostyle (PL) and ventrally to hypural 2 (H2), forming a V-shape in lateral view. Between them, three hypurals are positioned (H3-H5), with their bases lying just below the pleurostyle. The posterior tip of the notochord is covered by the opisthural cartilage (opc), which extends distally between the first dorsal procurrent and first principal caudal fin rays. Lateral to the posterior portion of the pleurostyle lies a short uroneural (UN) and dorsally a single median epural (E) which extends dorso-posteriorly.

### Ontogeny of the CC

The ontogeny of the compound centrum was analyzed through series of alcian blue and alizarin red cleared-and-stained specimens. A broad ontogenetic series extending from 4 mm SL pre-flexion larvae up to more than 30 mm SL adults was collected (Fig. 2c–h) and was contrasted with the available information about zebrafish development, including the sequence of appearance and ossification of elements [11, 13, 30, 31]. Discrepancies with previous works are highlighted below.

Early in development, the terminal portion of the notochord had a smooth surface, articulating ventrally with a series of well-developed cartilaginous haemal arches and hypurals, while flexing dorsally between hypural 1 and hypural 2 (Fig. 2c). After the vertebral centra mineralize and ossify over its surface, the notochord begins a widening at the intervertebral space between the preural centrum 2 (PU2) and the compound centrum (CC) that is accompanied by an amphicoelus or conic growth on the adjacent vertebrae (Fig. 2d–h). On the posterior side of the compound centrum (CC), the notochord does not considerably widen and the centra retained a more cylindrical instead of conic shape. Dorsally, a membranous pleurostyle initially had a narrow and contorted shape, extending over the dorso-posterior surface of the compound centrum and the notochord (Fig. 2d–e). However, over time, the pleurostyle widened ventrally over the notochord, reaching and sometimes covering the base of the hypurals 3 to 5 (Fig. 2f–h).

Ventrally, the parhypural and the hypurals underwent an extensive perichondral ossification process. The ossification of the parhypural began in the distal portion of the haemal arch and extended into the proximal portion of the spine, while the ossification of the hypurals began near its base and extended distally through the surface of each element (Fig. 2c–e). Among them, the parhypural and the hypural 1 each retained a large mass of unossified cartilage (or basiventral arcualia) near their base that articulated with the ventral portion of the compound centrum. The hypural 2 retained a remain of the basiventral as a small mass of cartilage on the anterior portion of its base (Fig. 2d) before eventually ossifying it perichondrally and fusing it with the compound centrum. Hypurals 3 to 5 did not retain a basiventral and completely ossified their bases early in development. Finally, as the pleurostyle ossification process grew ventrally and the hypural 2 ossification extended dorsally over the centrum, they fused at the posterior portion of the compound centrum to form the V-shaped connection between both structures observed in adults (Fig. 2f–h).

### Phases on the ontogeny of the CC

**Fig. 3:**
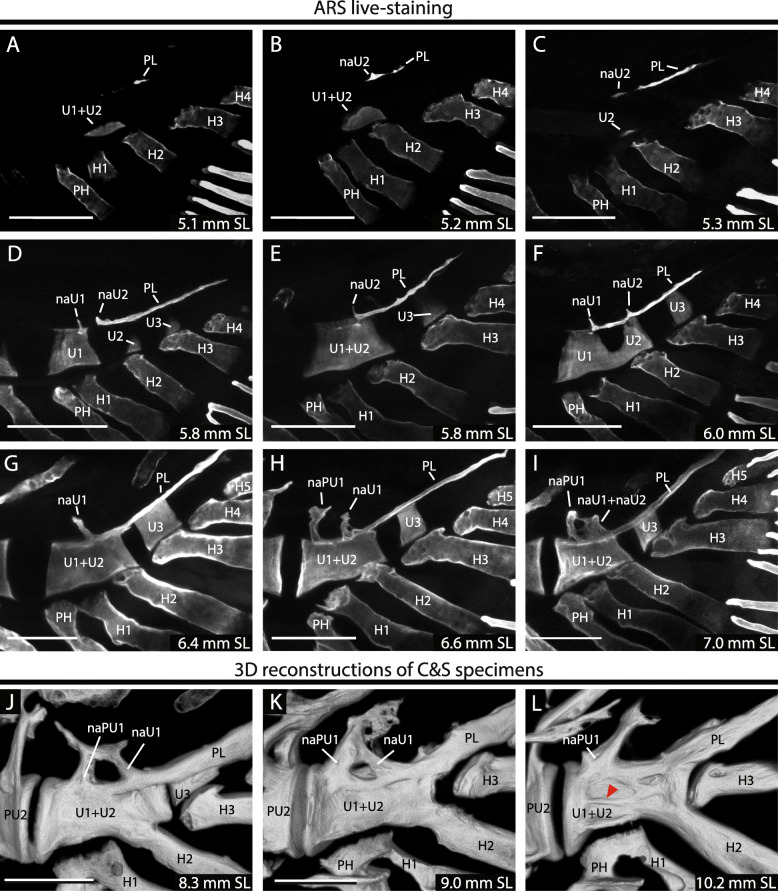
Mineralization of the compound terminal vertebra. **A–I**. Zebrafish larvae incubated in Alizarin red S and visualized through confocal microscopy. **J–L**. 3D reconstructions of cleared and stained individuals visualized through confocal microscopy. Red arrowhead points to the lateral middle ridge or crest. Terminology: cU1 + 2, ural chordacentrum 1 + 2; cU1–3, ural chordacentra 1 to 3; H1–5, hypurals 1 to 5; haPU1, haemal arch of the preural centrum 1; naU1-U2, neural arch of the ural centra 1 or 2; PH, parhypural; PL, pleurostyle. Scale bar: 100 μm

In order to understand the processes that occur during the development and assembly of the compound terminal vertebra, its ontogeny was divided into a series of three phases (Fig. 3): patterning, fusion and differential growth; this was complemented with an ontogenetic model that accompanies its description (Fig. 5). The **patterning** phase refers to the process by which the constitutive pieces or elements of the vertebrae appear during development, neural arches (na), the pleurostyle (PL), hypurals (H) and the vertebral centra from a previously unsegmented notochord (Fig. 5a–d). The **fusion** phase involves the interactions between these elements during ontogeny (Fig. 5e–f). And, the **differential growth** phase involves the changes that the already fused elements undergo throughout ontogeny, which involve the differential ossification of the anterior portion of the centrum, as well as, the distal growth of each of the fused elements, pleurostyle, neural arches and, certain parts of the compound terminal vertebrae, resulting in its asymmetrical growth (Fig. 5g–h).

### Patterning phase

The appearance of the caudal fin centra and their surrounding structures occurred mostly in larvae that had reached around 5 to 7 mm SL (Fig. 3a–i). Since the acid step, during the clearing-and-staining procedure, may demineralize the newly formed bone, we complemented this analysis by using Alizarin red S in vivo staining [45]. Using these procedures, we were able to distinguish the formation of ural centra and associated elements at earlier developmental stages than previously reported [30, 31]. Thus, in the following section, we dissect the different patterning processes. The interpretation of the identity of the elements follows the polyural interpretation, which involves the recognition of the segmental position of an element in relation to the neural arches and the hypurals.

### Patterning of the caudal fin centra

The first centrum appeared as early as 5.1 mm NL larvae adjacent to the base of hypural 2 (H2) or in between H1 and H2 (Fig. 3a–c). This centrum forms at the ventral portion of the notochord and grows dorsally in a crescent shape, until it surrounds the notochord and fuses dorsally with its contralateral portion to form a ring (compare Fig. 3d and f). A common variation was that two centra are formed instead of one, identified as ural centrum (U1^P^) and ural centrum 2 (U2^P^), which rapidly fuse into a single compound centrum (U1 + U2^P^) (Fig. 3f) and they do so from their ventral portion (Fig. 5a–c). Therefore, the single centrum observed in many specimens (Fig. 3c) could be caused by an earlier fusion of two centra, or by a variation in the patterning forming one centrum instead of two. Occasionally, these ural centra 1 and 2 (U1^P^ and U2^P^) may not fuse and remain as separate elements until adulthood, as previously shown [11, 30]. A preural centrum 1 (PU1) was never observed over the base of the parhypural (PH) in any of our specimens, as previously noted [11, 13], and the space left by its absence was instead filled by an anterior growth of U1^P^ or U1 + U2^P^ (Figs. 3d-f; 5b).

Posterior to ural centrum 2 (U2^P^) or the compound centrum (U1 + U2^P^), a third ural centrum formed over the base of hypural 3 (U3^P^), variably present in most specimens over 6 mm SL (Fig. 3d–i). Under a diural terminology, this centrum was previously identified as U2^D^ or U2^D^+ [30, 31]. As was the case for the first and second ural centra (U1 + U2^P^), this element grew dorsally in a crescent shape (Figs. 3d; 5b), surrounding the notochord and forming a ring (Fig. 3c–i). In our samples we never observed a distinct ural centrum forming posterior to the U3^P^, in contrast to previous reports [30]. However, considering the possibility of a posterior ural centrum, the zebrafish caudal skeleton may form up to four distinct ural centra, U1^P^, U2^P^, U3^P^ and U4^P^, with the most common pattern being the formation of an anterior centrum U1 + U2^P^ and a posterior U3^P^.

### Patterning of the neural arches and the pleurostyle

Dorsal to the compound centrum, as previously reported for zebrafish [13, 30], the pleurostyle plus a varying number of neural arches appeared (Fig. 5a–c). They arose almost simultaneously with the first ural centrum, with some specimens ossifying a pleurostyle before mineralizing the ural centrum or vice versa, therefore in contrast with previous research [30]. The identity of these elements was assigned based on their segmental position over the notochord with respect to the hypurals. The most common pattern observed consisted of two neural arches plus the pleurostyle, varying from one to three arches (Fig. 3).

The pleurostyle was the first epaxial element to appear over the notochord, in front of the bases of the hypurals H2 and H3 (Figs. 3a–c; 5a). Shortly after it appeared, it fused throughout with a separate ossification center forming above H2, interpreted here as the neural arch of the ural centrum 2 (naU2) (Figs. 3b–c; 5a). These elements were already fused in most specimens analyzed and only a few showed an unfused pleurostyle from the naU2 (Fig. 3c). When fused, it was commonly found that the pleurostyle developed a dorsal projection on its anterior portion, interpreted here as the remnant of this neural arch (Figs. 3d–f; 5b). As the pleurostyle grew posteriorly, another ural neural arch appeared above the hypural 1, interpreted as the neural arch of ural centrum 1 (naU1) (Figs. 3d–f; 5b). While initially independent, the neural arch of U1 (naU1) also fused with the pleurostyle, forming a compound F-shaped structure involving both ural neural arches and the pleurostyle (Figs. 3a–f; 5c–d). Later, at about 6.5 mm SL a third neural arch appeared anterior to the neural arch of U1 (naU1), occasionally located at the intervertebral space between the compound centrum (CC) and the preural centrum 2 (PU2), interpreted as the neural arch of the preural centrum 1 (naPU1) (Fig. 5f). The presence of this arch was highly variable between specimens and even between the left and right sides of the same individual and, once formed, this arch fused distally with the neural arch of ural centrum 1 (Figs. 3i; 5g–h). In summary, the epaxial elements of the compound centrum, including the neural arches of PU1, U1 and U2 (naPU1, naU1 and naU2) and the pleurostyle, appear and fuse sequentially from posterior to anterior, with a high intraspecific variation, with only the neural arch of U1 (naU1) and the pleurostyle consistently present in all specimens analyzed.

### Fusion phase of the compound terminal vertebrae

Once the elements of the compound terminal vertebrae were patterned, during further development they began to interact with each other through a series of fusions. By using confocal images and 3D models of older cleared and stained individuals, we characterized the series of fusions between these structures (Fig. 3j–l). As previously detailed, the first series of fusions occurred between the ural neural arches and the pleurostyle (Fig. 3a–i), and between ural centrum 1 and ural centrum 2 (U1^P^ and U2^P^) (Figs. 3d–F, 5a–b).

The next major fusion occurred between the compound centrum (U1 + U2) and the ural neural arches plus the pleurostyle, fusing through the anterior portion of the centrum on specimens between 6.5 and 8 mm SL (Figs. 3j–k; 5e). Once established, this fusion advanced in an antero-posterior direction, fusing all the ventral surface of the ural neural arches and pleurostyle with the dorsal portion of the centrum (compare Figs. 3j, k and l; 5d–h). In addition, the distal portions of naPU1 and naU1 fused distally between each other, forming an O-shaped neural arch (Fig. 5h). Posteriorly, the pleurostyle grew ventrally over the dorsal surface of the notochord, partially covering the lateral surface of the ural centrum 3 (U3^P^) (Fig. 5e–g). At these same stages, on the ventral side of the compound centrum, the hypural H2 fused with the compound centrum (Figs. 3j–k; 5f).

### Differential growth of the compound terminal vertebra

The last step in the formation of the compound terminal vertebra was the differential growth of the pleurostyle, hypural 2, neural arches and autocentrum observed in the sequence of individuals that ranged from 10, 18 to 31 mm SL (Fig. 2). Once the pleurostyle and the hypural 2 were fused to the centrum, they began to develop a thick U-shaped connection over its posterior portion (Figs. 3l; 5g–h). This connection grew thicker and started to grow posteriorly throughout development, eventually covering the whole surface of U3^P^ that had become incorporated in a cavity beneath the pleurostyle (Fig. 5h). On the lateral surface of the compound centrum, a longitudinal middle ridge developed, which grew laterally leaving dorsal and ventral cavities in the surface of the centrum (Figs. 3l: red arrowhead; 5h). Dorsally, the fused ural neural arch grows dorsally into a short membranous spine, with the foramen formed by the fusion of naPU1 and naU1 becoming progressively smaller as the vertebra grew, until eventually disappearing (Fig. 5h).

### Ossification of the autocentra

Since the vertebral centrum in teleosts forms through two different processes, we distinguished them by i) the mineralization of the fibrous or middle layer of the notochord (chordacentrum), or ii) the ossification on the outside of the notochordal sheath (autocentrum) by the presence of osteoblasts covering their surface. This distinction was achieved by complementing the analysis of alizarin red-S live stained larvae with zebrafish carrying the transgenic marker *sp7*:*nu*GFP, which express the green fluorescent protein in osteoblast nuclei [44]. Thus, a vertebral centrum stained with alizarin red but lacking osteoblasts on its surface indicates a mineralization of a chordacentrum, while their presence would indicate the ossification of an autocentrum.

**Fig. 4:**
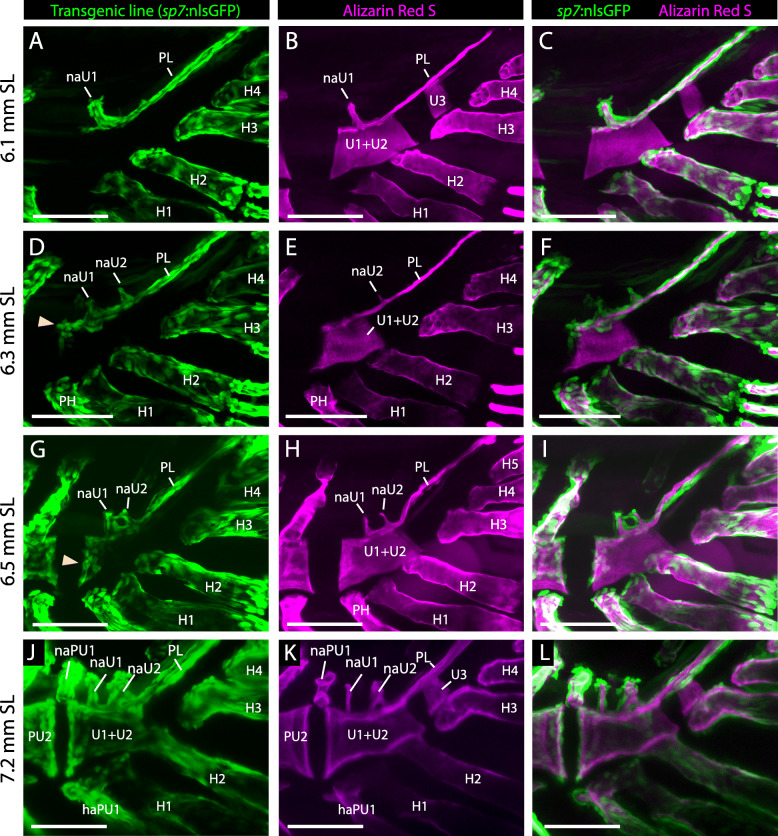
Patterning of the autocentrum. Confocal images of the osteoblast transgenic reporter *sp7*:nlsGFP, counterstained with the bone marker alizarin red S, were taken at different stages of zebrafish development to show the conformation of the compound centrum. Terminology: aut, autocentrum; cU1 + 2, ural chordacentrum 1 + 2; H1–5, hypurals 1 to 5; haPU1, haemal arch of the preural centrum 1; naPU1-U1-U2, neural arch of the preural centrum 1 or ural centra 1 or 2; PL, pleurostyle. Scale bar: 100 μm

**Fig. 5:**
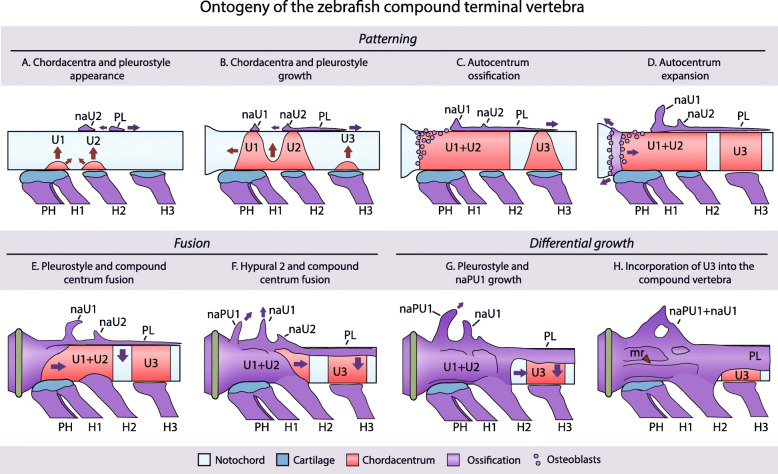
Zebrafish terminal vertebra assembly model. The model considers eight steps on the formation of the zebrafish terminal vertebra, divided between phases of patterning, fusion and differential growth. Arrows indicate direction of growth. The red arrowhead on the last panel points to the lateral middle ridge or crest. Terminology: H1–3, hypurals 1 to 3; mr, middle ridge; naPU1, neural arch of the preural centrum 1; naU1–2, neural arch of the ural centra 1 and 2; PH, parhypural; PL, pleurostyle; PU2, preural centrum 2; U1–3, ural centra 1 to 3; Un, uroneural

We found that, in larvae that had reached 5 to 6 mm SL, the caudal fin centra were exclusively formed as chordacentra, observed by the lack of osteoblasts on their surface (Fig. 4a–c). By contrast, the membranous ossification of the pleurostyle and neural arches, as well as the perichondral ossification of the hypurals and the parhypural, were completely covered by osteoblasts. In larvae measuring 6.3 to 6.5 mm SL, a group of osteoblasts had colonized the anterior border of the compound centrum (U1 + U2; Fig. 4d–i). This colonization seemed to advance over the surface of the compound centrum from anterior to posterior, almost reaching the posterior border of the centrum in 7.2 mm SL larvae (Figs. 4j–l; 5c–f). At the same stages, and in contrast to the anterior border, the posterior border of the centrum (U1 + U2) was not colonized by osteoblasts (Fig. 4a–l). Therefore, there was a remarkable asymmetry in autocentrum formation between the anterior and posterior borders of the compound centrum, which is, in turn, also different compared to more anterior vertebrae. Furthermore, the ural centrum 3 (U3) was never colonized by osteoblasts in any stage analyzed (Fig. 4a–l), as it remained only as a ring-like chordacentrum. Therefore, the anterior border of the compound centrum is the last autocentrum to form along the axial skeleton of zebrafish, with the posterior border of the compound centrum (U1 + U2^P^) and the ural centrum 3 (U3^P^) forming only as chordacentra.

## Discussion

### Chordacentra development

The formation of two concentric cylinders, the first within and the second outside of the notochordal sheath seems to correspond to a generalized pattern during the formation of the vertebral centra, which was already present in stem teleosts since the Jurassic, including †*Leptolepis coryphaenoides* and more advanced teleosts [8, 10–12, 27, 36, 46]. The inner cylinder, or chordacentrum [6, 47] is present among neopterygians (teleosts plus holosteans) and forms through a mineralization of the notochordal middle layer, known as the fibrous layer or tunica media [48, 49], by the activity of chordoblasts lining the notochord inner epithelium, and is thus independent of osteoblasts [32–35, 50, 51]. Most chordacentra appear to follow an antero-posterior direction in zebrafish [33, 34]. However, as in other teleosts, including salmonids, *Hiodon* and *Elops* [9, 10], the zebrafish ural chordacentra do not follow this antero-posterior wave of chordacentrum formation with U1 + U2^P^ and U3^P^ forming before the more anterior preural centra.

Within the caudal fin skeleton, each chordacentrum emerged from the ventral surface of the fibrous layer of the notochord and grew dorsally in a crescent shape, acquiring a ring-like appearance after surrounding the notochord (Fig. 3a–f) [11, 51]. This mode of growth was first proposed as a generalized feature among basal teleosts [3] and later was shown particularly for zebrafish [51]. In recent years the mechanisms by which the notochord intrinsically segments and patterns into chordacentra has made significant progress [33, 34]. Interestingly, the notochord seems to pattern into vertebral and intervertebral segments, via the activation of the Notch signaling pathway, in a manner that is independent from somitic segmentation [33, 34]. However, it is not clear whether the patterning of the chordacentra may have distinct regional variation that may explain how the most posterior portion develops outside of this antero-posterior wave, which may suggest that the patterning of caudal fin vertebrae answer to a different developmental module. Further works focusing on the molecular aspects of caudal fin formation will improve our understanding of this variation along the axial skeleton.

Experimentally, similar types of fusion between adjacent chordacentra may be induced by two mechanisms: first, by altering the notochordal patterning through a manipulation of the Retinoic Acid (RA) or Notch signaling pathways, as shown in in zebrafish [33, 44, 52]; or second, by a conditional ablation of osteoblasts during the formation of autocentra, as demonstrated in medaka (*Oryzias latipes*) [53]. Thus, although the particular mechanism by which these adjacent ural centrum 1 and 2 fuse in zebrafish remains unexplored, it may reflect some regional variation on RA or Notch signaling during early development, or in turn, as in medaka, because of the absence of an autocentrum forming between both centra which might induce the ontogenetic fusion of both chordacentra.

### Autocentra development

In contrast to the inner cylinder, the outer cylinder or hourglass-shaped autocentrum [6] appeared later during the evolution of teleosts, present since †*Leptolepis coryphaenoides* and more derived teleosts [2, 5]. This centrum ossifies directly from the perichordal mesenchyme outside of the notochord, by the activity of osteoblasts lining the outer surface of a chordacentrum [33, 35–37, 54–56]. Once colonized, autocentral growth is achieved through the ossification of the active anterior and posterior edges of the centrum [35, 37, 56] or the so-called vertebral endplates [57], which together with the gradual widening of the intervertebral disc, gives the hourglass shape to the centrum.

This mechanism of anterior and posterior autocentral growth seems to be generalized along the zebrafish axial skeleton [33] and is tightly associated with the growth of the adjacent intervertebral discs. In fact, Grassi (1883: translated p.320, 341) was the first to describe this link during the development of cyprinids, salmonids and in *Esox*, where he stated that the “chordal substance present over the intervertebral disk extends distally and folds over the surface of the adjacent centrum, forming a thin sheath over it”. François (1966) observed a similar association between the autocentrum and the intervertebral disc in *Salmo,* describing the autocentrum of each vertebra forming from two separated rings of bone over the anterior and posterior edges of each chordacentrum before fusing medially. More recently and supporting this association, it has been shown in medaka that osteoblast progenitors that form the autocentrum derive from somitic segmentation and reside within the intervertebral discs before colonizing the surface of the chordacentra to ossify the autocentra [37, 58].

Overall, this relationship between the intervertebral discs (IVDs) and autocentral ossification was consistent with our analysis of the zebrafish caudal fin skeleton, where the last intervertebral disc was located between the preural centrum 2 and the compound centrum (Fig. 2), and the last autocentrum formed on the anterior edge of the compound centrum (Fig. 4); there was no formation of IVDs nor autocentra in the ural region. Further work will help to understand the relationship between IVDs and autocentra, and their absence in the most posterior portion of the caudal skeleton, as well as how osteoblasts that derive from somitic segmentation become re-specified to follow, instead, the notochordal segmentation of chordacentra.

### Evolution of the compound terminal vertebrae among ostariophysan fishes

Gosline [19] highlighted the morphological similarity between the caudal skeleton of cypriniforms, characiforms and siluriforms as they have a single terminal centrum fused to an anterior pair of uroneurals (pleurostyle) and the hypural 2, with an upright neural arch (Fig. 1). Later, when Rosen & Greenwood [59] included the gonorynchiforms to the ostariophysans, they assumed that the ostariophysan ancestor must have also shared a caudal skeleton with a compound terminal vertebra (as in *Chanos*; Fig. 1). However, since fossil gonorynchiforms such as †*Tharrhias* and †*Dastilbe* lack a consolidated caudal skeleton, Fink & Fink [15, 28] interpreted that the compound terminal vertebra was a homoplastic character that must have evolved independently in gonorynchiforms and otophysans. They also included the compound terminal centrum as one of the main otophysan synapomorphies (character 110), which was characterized as the fusion between the first preural centrum and two ural centra (PU1 + U1^D^ + U2^D^). However, later authors disagreed on this shared composition of the compound terminal centrum among ostariophysans, with some of them excluding the U3^P^ from the compound centrum (see below) while others questioning the presence of a PU1 during development [11, 13].

### Compound centrum

The zebrafish compound centrum develops from one or two chordacentra that mineralize in front of hypurals 1 and 2 and fuse early in development (U1 + U2^P^). This view contrasts with the previous *diural* interpretation of the caudal skeleton for zebrafish, where these centra were interpreted as the PU1 and the U1, instead [30, 31]. This has been thoroughly discussed by Schultze and Arratia (2013) and Wiley et al. (2015).

A single ural centrum forming above hypurals 1 and 2 is the most common pattern across basal teleosts, including †*Leptolepis*, †*Ascalabos*, †*Tharsis, Elops*, *Hiodon*, salmoniforms, alepocephaliforms and clupeomorphs [11, 27]. However, a common variation from this pattern involves the formation of two independent U1^P^ and U2^P^ centra that, as in zebrafish, are occasionally observed in the salmoniforms *Thymallus* and *Oncorhynchus* [10] and in the fossil osteoglossomorphs †*Asiatolepis* and †*Lycoptera* [11]. Hence, an occasionally unfused U1^P^ and U2^P^ may suggest a vestige or atavism of this character from a previous *polyural* pattern in teleosts, as this is the condition present in holosteans and stem teleosts such as †*Pholidophorus*, †*Eurycormus* and †*Catervariolus* [11]. Further analyses showing the ontogeny and intraspecific variation in other teleosts will help to uncover the pattern and frequency on the formation of separate U1^P^ and U2^P^.

### Absence of a preural centrum 1

Our results fully support the lack of PU1 in zebrafish [11, 13], also reported for the consolidated terminal vertebra present in the euteleost *Mallotus* [12], and the siluriform *Ictalurus* [60]. By contrast, a PU1 is present among all studied caudal skeletons with unconsolidated terminal vertebrae, such as salmoniforms [10], the alepocephaliform *Talismania* [27] and some clupeomorphs [11, 61]. Thus, the distribution of this character seems to be related with the acquisition of a consolidated caudal skeleton. However, only further studies on different taxa with consolidated caudal skeletons may help to establish this relationship, as well as if there is a phylogenetic signal associated with the loss of a PU1 [13].

### Autocentrum of the compound centrum

Autocentral growth limited to the anterior border of terminal vertebra is a widespread character in teleosts that have a consolidated caudal skeleton, such as ostariophysans, argentiniforms, and osmeriforms [11, 16]. It also marks the limit of the most posterior intervertebral articulation, located between the compound centrum and the preural centrum 2 (PU2). By contrast, in basal teleosts such as *Elops*, *Hiodon*, *Thymallus*, *Sprattus* and *Alepocephalus* (Fig. 1), the preural centrum 1 (PU1) and the first ural centrum develop complete hourglass-shaped vertebrae, with intervertebral discs located more posteriorly, which may indicate a more flexible terminal portion of the axis. Thus, it seems that the acquisition of a compound terminal centrum correlates with an anterior displacement in the formation of the last autocentrum and intervertebral disc during development.

### Third ural centrum or U3^P^

Some of the earliest works that analyzed ostariophysan larvae excluded the U3^P^ from the compound centrum, which was interpreted instead as a reduced or vestigial structure that “never advanced beyond the chordacentrum stage” [62] and “comes to lie in the cavity on the posterior face of the compound centrum” [22]. This view was further supported by later analyses in the siluriforms *Nematogenys* and *Trichomycterus*, which showed a third ural centrum U3^P^ (or U2^D^) enclosed beneath the pleurostyle and unfused to the compound centrum [63]. Additional ural centra (U3^P^, U4^P^ and U5^P^) has been reported for a variety of extant teleosts outside ostariophysans, including some elopomorphs, clupeomorphs and salmonids [8, 10, 11, 46, 61]. Our results fully support that the zebrafish ural centrum 3 remains as an unfused chordacentrum, in agreement with Schultze and Arratia (2013) who stated that “the centrum (U3^P^) abuts the compound centrum but it does not fuse to it”. Previous studies in *Danio*, however, have included the U3^P^ as part of the compound centrum [13, 30, 31]. Bensimon-Brito et al. (2012) interpreted that the second ural centrum fuses to the compound centrum through a dorsal perichordal ossification outside of the notochord, interpreted as an autocentrum, while its chordacentrum remained separated and unfused to the compound centrum (their Fig. 4). However, in our analysis, no autocentral ossification is formed in this centrum, but instead in the same position is located the pleurostyle, which grows from dorsal to ventral over the notochord, and would be similar in appearance in histological sections. Finally, considering that the stem ostariophysan †*Tischlingerichthys* [4] and the stem otophysans †*Chanoides* and †*Nardonoides* [64], also have a U3^P^ unfused to the compound centrum, the presence of a vestigial and unfused U3^P^ formed only as chordacentrum may represent the plesiomorphic condition for otophysans.

### Hypural 2

*Danio* shares the basal condition common to cypriniforms of hypural 2 fused to the compound centrum [15]. This fusion occurs after the perichondral ossification of hypural 2 reaches its base and contacts the centrum, where it fuses to the autocentral ossification of the compound centrum and the membranous ossification of the pleurostyle. This compound centrum/hypural 2 fusion seems to be a homoplastic character that has been convergently acquired among otophysans [15], gonorynchids [65] and clupeomorphs [66], while it is absent in *Chanos* and most kneriids among gonorynchiforms [65], as well as in alepocephaliforms [27] and in the stem ostariophysan †*Tischlingerichthys* [4] and in the stem otophysans †*Chanoides* and †*Nardonoides* [64].

### Neural arch of the compound centrum (naCC)

As they did for other cyprinids, Sanger and McCune (2002) interpreted that a single neural arch over the compound centrum was the common pattern in *Danio*, although they recognized an occasional doubling of this arch. In ontogenetic studies, Bird and Mabee (2003) recognized a single neural arch over the compound centrum, while, Bensimon-Brito et al. (2012) recognized the presence of three rudimentary neural spines over the centrum. On the other hand, Schultze and Arratia (2013) and Wiley et al. (2015) expressed reservations about identifying these elements, since the arches instead of forming as endochondral elements, as in more basal teleosts, form as a membrane bone without a cartilaginous precursor. Further yet, Wiley et al. (2015) stated that due to this significant change in the developmental process, these neural arches could not be homologized with the arches of more basal teleosts. The scarce current information about the ontogenetic development of these structures in stem teleosts refrains us to elaborate further on this subject. However, due to the presence in other cypriniforms of similarly positioned neural arches but with cartilaginous bases, including the cyprinid *Luxilus* [27], these neural arches might be tentatively interpreted sequentially as the neural arch of the preural centrum 1, ural centra 1 and 2 due to their location, pending further analyses in the developmental processes of the neural and haemal arches formation in other teleosts.

### Neural arch of the preural centrum 1 (naPU1)

One of the most variable elements within the zebrafish caudal skeleton is the position and growth of the neural arch of the preural centrum 1 (naPU1). As noted by Wiley et al. (2015), this arch was anteriorly displaced in some specimens up to the intervertebral space between the preural centrum 2 (PU2) and the compound centrum (Fig. 4j–l). However, this arch was normally positioned in the anterior portion of the compound centrum in front to the parhypural (Fig. 3g–i), where it fuses distally to naU1.

An anterior displacement of the naPU1 is a common variation found among basal teleosts, including *Elops* [8], *Hiodon* [8, 11, 67], in the salmonids *Oncorhynchus*, *Salmo*, *Thymallus*, *Salvelinus*, *Cristivomer*, *Prosopium* and *Coregonus* [10, 11, 46, 68], in the osmeriforms *Salangichthys* and *Retropinna* [68] and in the alepocephaliforms *Xenodermichthys*, *Searsia*, *Holtbyrnia* and *Talismania* [27, 69, 70]. Among ostariophysans, it is also observed in the cypriniforms *Catostomus*, *Luxilus* and *Moxostoma* [11, 27, 71], besides *Danio* (Fig. 4j–l) [13, 30]. In all these groups, the anterior displacement of this arch correlates with the dorsal flexion of the notochord, which could stress the dorsal elements by pulling them close together. In addition, the position of this arch also correlates with its distal elongation; since, when anteriorly displaced, it develops as a more elongated arch resembling the naPU2, while when patterned regularly over the parhypural, it develops as a smaller element resembling the neural arch of the ural centrum 1 (naU1). Thus, the position of the neural arch of the preural centrum 1 (naPU1) it is an important character that should be considered when analyzing the intraspecific variation of the caudal skeleton of teleosts.

When patterned normally in *Danio*, the neural arch of the preural centrum 1 (naPU1) fuses distally to the neural arch of the ural centrum 1 (naU1), leaving a foramen in between, and thus forming a single neural arch over the compound centrum. This fusion is a common feature among ostariophysans, including *Chanos* [61] and the siluriforms *Gagata* and *Trichogenes* [72]. On the other hand, in some characiforms the neural arch of the preural centrum 1 (naPU1) do not fuse with the ural neural arches, including *Prochilodus, Salminus* and *Gymnocharacinus* [15, 73, 74] and it is also a common variation among catostomids [62, 71]. The presence of a single neural arch over the compound centrum is the widespread character among ostariophysans, and therefore, if two independent neural arches form over the compound centrum, as in *Danio*, the fusion between them would probably represent the primitive condition, reversed in some characiforms and catostomids, that retain them as separated. Interestingly, a similar fusion between naPU1 and naU1 is also observed in the alepocephaliforms *Searsia*, *Holtbyrnia* and *Maulisia* [69, 70], and therefore through further ontogenetic and phylogenetic analyses this fusion might resolve as a synapomorphy at some level of Otomorpha.

### Ural neural arches (naU1 and naU2)

The presence of two ural neural arches forming over the compound centrum before fusing to each other to form a single neural arch in adults has been reported previously during the ontogeny of some ostariophysans. Among them, some *Chanos* specimens (gonorynchiforms) form two cartilaginous ural neural arches (naU1 and naU2) plus the neural arch of the preural centrum 1 (naPU1) before fusing to each other and ossifying perichondrally to form a single neural arch in adults [61]. Among cypriniforms, *Catostomus* and *Luxilus* form either one or two ural neural arches with a cartilaginous base, that ossify either through a perichondral or membranous ossification, which fuse to form a single neural arch in adults [11, 27]. In the characiforms *Salminus* and *Gymnocharacinus* more than one ural neural arch is observed, with all of them fusing and forming a single structure with the pleurostyle [73, 74]. Thus, the occasional presence of two ural neural arches that fuse early in development to form a single structure might be a plesiomorphic character for ostariophysans.

### Pleurostyle

The pleurostyle was originally defined as the paired dorso-posterior process of the preural centrum 1 [21] to include a similar structure observed in the adult caudal fin skeleton of clupeomorphs and ostariophysans (Fig. 1). However, since it is absent in intermediate taxa such as extant alepocephaliforms, the basal clupeiform *Denticeps* and in fossil stem clupeomorphs, it seems to have been convergently acquired [27]. Even among ostariophysans, there is still no clear consensus about the homology of the pleurostyle since fossil gonorynchiforms such as †*Tharrhias* and †*Dastilbe* lack a pleurostyle and instead have an unfused elongated uroneural at this position. Further yet, it is not clear if the pleurostyle develops as a modification of the same ural neural arch in all these groups due to the relative lack of ontogenetic information [11, 13]. In zebrafish, the pleurostyle patterns as a paired and independent membranous ossification on the dorsal surface of the notochord posterior to the neural arches of the ural centra 1 or 2 (naU1 or naU2) (Fig. 3a–d). It grows posteriorly surrounding the notochord, while anteriorly it sequentially fuses to the naU2 (when present), the neural arch of the ural centrum 1 (naU1) and the compound centrum.

Traditionally, the pleurostyle has been considered a modified uroneural that lost its anterior tip and became fused to the compound centrum [11]. Among ostariophysans, the cypriniforms *Luxilus*, *Catostomus* and *Moxostoma*, develop a pleurostyle located posteriorly to the first (naU1) or to the second ural neural arch (naU2) [11, 27, 71]. A similar pattern is observed in the gonorynchiform *Chanos*, where the pleurostyle forms posteriorly to either the naU1 or the naU2 [11, 61]. In the siluriforms *Ictalurus*, *Gagata* and *Trichogenes*, the pleurostyle forms posteriorly to naU1 [60, 72], while in the characiform *Salminus*, there seems to be at least three ural neural arches anterior to the pleurostyle [74]. Thus, considering the variation in the number of ural neural arches anterior to the pleurostyle, two options are available to understand the evolution of this structure among ostariophysans: either 1) the pleurostyle develops from *different* segments or modified neural arches in ostariophysan subgroups and, therefore, is *not* homologous and was independently acquired in each group, or 2) the pleurostyle develops from a modification of the *same* segment or uroneural in all these groups, and it is the number of ural neural arches anterior to it that changes. Our results favor the second scenario, since we observed a high variation in the number of ural neural arches. However, to establish the homology of the pleurostyle would not only require that the pleurostyle develops from the modification of the same segment, but also that it shares a conserved pattern of fusion to the centrum from their last common ancestor.

Few studies have detailed the patterns of fusion of the pleurostyle during development. In the cypriniform *Moxostoma* [71] and in the characiform *Salminus* [74] the pleurostyle fuses with the ural neural arch (es) *before* fusing with the compound centrum, as in zebrafish. In contrast, in the stem otophysans †*Chanoides* and †*Nardonoides* [64] and in the stem ostariophysan †*Tischlingerichthys* [4], the pleurostyle does not fuse to the neural arches but instead fuses to the centrum. Such distinction suggests that the pattern of fusion between the pleurostyle and the ural neural arch (es) is characteristic of extant otophysans and may reflect a synapomorphy for the crown group.

Only further ontogenetic and phylogenetic studies, particularly at the base of ostariophysans and otomorphs, will help to unveil the homology and evolution of the pleurostyle. We suggest that separating its ontogeny into patterning and fusion will help to uncover whether the pleurostyle present in clupeomorphs, gonorynchiforms and ostariophysans arose from the modification of the same segment and which relationships it has with the uroneurals present among other teleosts.

## Conclusion

The assembly of the zebrafish terminal vertebra involves a complex series of interactions during patterning, fusion and differential growth of each of its elements, a remarkably different process to all other vertebrae along the axial skeleton. Developmentally, different bone mineralization processes interact, including the formation of a chordacentrum, autocentrum, the membranous ossification of the neural arches and pleurostyle and the perichondral ossification of the hypurals. In addition, it is also a transitional zone between the preural and ural regions, that involves the formation of an intervertebral disc and an autocentrum anteriorly, but absent in the ural region, which instead is formed only as chordacentrum. Therefore, this region would be highly interesting to study in further developmental studies focused on elucidating the interactions between the distinct patterns of centrum formation during development.

From an evolutionary viewpoint, drastic changes in recent years concerning the phylogenetic classification of modern teleosts have defied longstanding morphological hypotheses that supported some of the major clades [75]. Therefore, the need for a detailed comparative analysis of embryos and adult teleosts has become one of the major challenges towards establishing the morphological support for each of the subgroups of extant and fossil teleosts. In this context, the caudal fin skeleton has a long tradition of being a trait that supports some of the major groups of teleosts, and it is now one of the most detailed and studied structures in this group [2]. We now know that the acquisition of a consolidated terminal vertebra fused to the first uroneural was independently acquired several times in different groups of teleosts, which seems to follow a general trend towards a more robust and consolidated caudal skeleton apt for swimming propulsion. By distinguishing the position where each element develops and their interactions through time and space, it is possible to understand their homology to similar structures found in other teleosts and trace their evolution. In this context, the distinction between a polyural and a diural nomenclature was of major importance, due the phylogenetic implications over understanding the homology of similar structures over distantly related groups. Several questions remain, however, including the variation in the patterning of the neural arches and pleurostyle formation among ostariophysans, or the convergence of characters during the consolidation of the caudal skeleton of teleosts.

## Data Availability

Zebrafish specimens used for comparative anatomical analyses during this study are included in this published article (see section on Material and Methods). Raw confocal images of specimens generated in this study are available from the first author upon request.
